# English Doctors Abroad

**Published:** 1895-09-28

**Authors:** 


### The Hospital

AN INSTITUTIONAL JOURNAL OF

The Medical Sciences and Hospital Administration,

WITH

AN EXTRA NURSING SUPPLEMENT.

VOL. XVIII.—No. 470.] [SEPT. 28, 1895.

### ENGLISH DOCTORS ABROAD.

THAT the English are a roving, wandering race, given greatly to colonisation, has long since passed into a truism, and it must to many be a matter of surprise that a larger number of British medical practitioners are not found practising among the hosts of English-speaking people scattered over the globe. In regard to the numerous English settlements which exist in foreign countries there are many reasons for this. One difficulty is that of the language, another the examinations which must in so many places be passed before practice is permitted, and a third is the fact that the English settlements in foreign countries have not been the growth of a day, and are generally strongly leavened with old residents who know all the ins and outs of the place, and that these sojourners in strange climes avail themselves of the best local medical talent, just as they do of the best of everything else which the place provides. The mere fact, then, of being an Englishman must by no means be looked upon as likely to be a passport to medical practice amongst the English settled in foreign countries.

The examinations are a serious drawback. In Austria, Denmark, Holland, Germany, Greece, Portugal, Roumania, Russia, Norway, Spain (including its colonies), Sweden, Switzerland, and even Turkey, a State examination must be passed. In some of these countries however, such as Russia and Spain, the holder of a degree of M.D. from a university of high repute may obtain some modification of, or exemption from, the examination through the intervention of the British Ambassador.

In France the degree of M.D., obtained by examination, is indispensable; but exemption from certain limited portions of the curriculum and examination may be obtained by holders of British diplomas, at the discretion of the Minister of Public Instruction. In Italy, practice among foreigners is allowed to possessors of English diplomas, and in Monaco an English M.D. degree and certain English diplomas are accepted. In most of the countries in South America, and in a good many parts of the United States, an examination has to be passed, as also is the case even in British Columbia and in Ontario, Canada.

It is clear then that it is by no means open to an English medical man to practise where he pleases, unless indeed he pleases to practise in one or other of the British Colonies or Dependencies with the exception of the two above named. There is, however, under our own flag a very large field, and with the rapid development of the scientific side of medicine this field ought for some years to come to absorb a large number of well-educated medical men. We say "well educated" advisedly. The time is past when the second class man, the crank, the failure of the schools, could comfort himself with the idea that what would not go down in England was good enough for the Colonies, and that at the worst he could emigrate. The Post Office and the Press have brought the whole of Greater Britain into intellectual touch with the mother country. Almost every Colony is within six or seven weeks of London. What is thought and talked of here is thought and talked of there, often indeed with much more inquisitiveness and far less respect or deference to authority than at home, so that the man who rests satisfied with his diploma and dogmatises on the strength of it is soon found out. In fact, it is the same with medicine as with everything else. It is not the village lout who makes the good emigrant, but the capable handy man. It is not the backward student who has been lifted into the profession by the crammer who makes the good colonial doctor, but the man who would also, although probably after a longer time of waiting, succeed in England—the man who has mastered the principles of medicine and can apply them amid every variety of surroundings.

For such men, however, there can be no doubt that in many foreign countries, but especially in many British colonies, there is a good opening. The advantages of Colonial practice must not be entirely measured by the amount of Colonial fees. There is the freer life, often the better climate, and there is also the lessened anxiety about the future of sons and daughters, for however common are the failures of young people who emigrate—largely in consequence of defective training—the children of colonials brought up in colonial ways have good chances. It is not everyone, however, who should emigrate. Far too often men have gone out who have already failed, either in health, in morals, or in practice.

These are not likely to succeed. Nor should men whose mental tendency is to rely on others for advice and help go where neither help nor advice can be secured. But for the well-trained, self-reliant man, well versed in his profession, and endowed with health and a love of outdoor life, practice in some of our colonies certainly offers more attractions than do many forms of general practice in the slums and alleys of our own more crowded land.

